# COVID-19 and the Concept of Thrombo-Inflammation: Review of the Relationship between Immune Response, Endothelium and Coagulation

**DOI:** 10.3390/jcm12237245

**Published:** 2023-11-23

**Authors:** Emmanuel de Maistre, Philippe Savard, Pierre-Gregoire Guinot

**Affiliations:** 1 Haemostais Unit, Dijon University Hospital, F-21000 Dijon, France; philippe.savard@chu-dijon.fr; 2 Department of Anesthesiology and Intensive Care, Dijon University Hospital, F-21000 Dijon, France; pierregregoire.guinot@chu-dijon.fr

**Keywords:** COVID-19, cytokine storm, complement activation, vasculopathy, hypercoagulopathy, platelet activation, anticoagulation treatment

## Abstract

COVID-19, caused by the SARS-CoV-2 virus, has revealed a complex interplay between inflammation and coagulation, leading to the emergence of the concept of thrombo-inflammation. This concept recognizes that COVID-19 is not solely a respiratory illness, but a systemic disease with significant vascular and hematological components. COVID-19 is associated with an unusual prothrombotic state, with intense endothelial activation leading to vasculopathy, cytokine storm, complement system activation and a hypercoagulability state (the activation of platelets and the coagulation cascade, impaired fibrinolysis). The aim of this review is to discuss the different pathological pathways described in COVID-19 that lead to thromboembolic events. Widespread vaccination and post-COVID-19 immunization allows control over the severity of this pandemic. A better understanding of the pathophysiology of COVID-19 can improve the management of frail patients who are hospitalized in intensive care units.

## 1. Introduction

In March 2020, the World Health Organization (WHO) declared coronavirus disease 2019 (COVID-19) a pandemic. COVID-19 is strongly associated with a high incidence of thromboembolic complications, with pulmonary embolism often taking a leading role and being responsible for sudden deaths, particularly during the initial wave of COVID-19 [1,2]. Acute lung injury induces endothelial damage, triggers the coagulation cascade, and leads to in situ pulmonary thrombosis [3]. In COVID-19, there is an endothelial tropism exhibited by the SARS-CoV-2 virus, impacting not only the respiratory system but also other organs. This phenomenon underlines the severity of this viral pathology, with a key role played by the ACE2 receptor.

**Figure 1:**
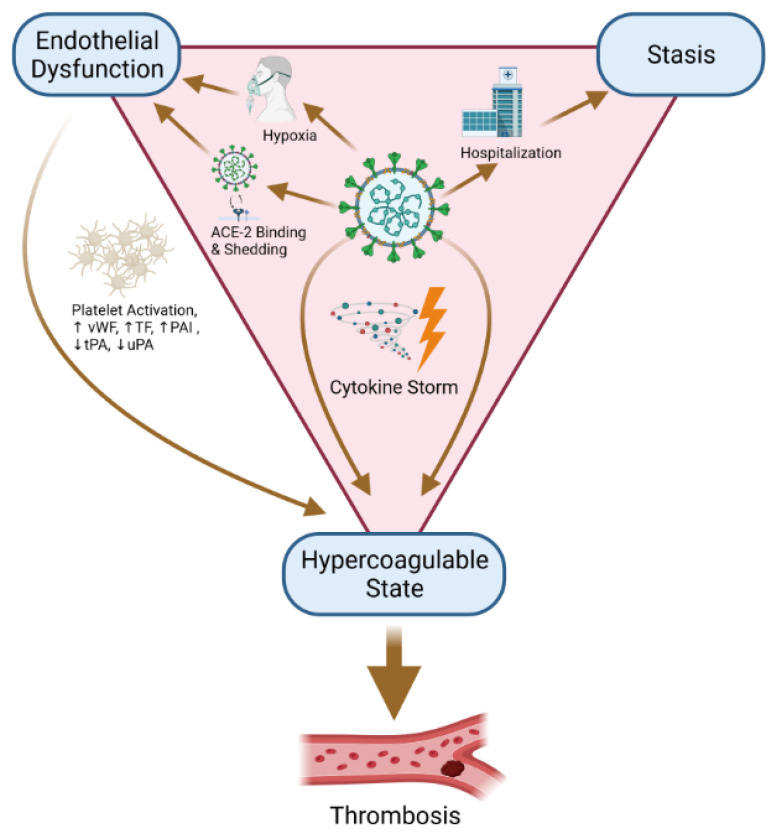
Virchow triad: stasis, hypercoagulable state and endothelial dysfunction (Created with BioRender.com, accessed on 31 October 2023).

There is a complex interplay between inflammation and coagulation in COVID-19. Typically, patients with severe sepsis can develop coagulopathy due to an intense inflammatory syndrome, including the local expression of tissue factor, the initiation of the coagulation cascade and thrombin generation, ultimately resulting in disseminated intravascular coagulopathy (DIC). In COVID-19, we observe a distinct coagulopathy, which is prothrombotic in nature and contributes significantly to morbidity and mortality [2]. In fact, COVID-19 is primarily associated with endothelial disease (or vasculopathy) and secondarily with coagulopathy, characterized by impaired fibrinolysis, which is linked to an excessive immune response (cytokine storm) [4]. We have the three factors of the famous Virchow’s triad contributing to the formation of thrombosis: venous stasis (immobilization, hospitalization), hypercoagulability (with biomarkers such as D-dimers) and vascular injury (Figure 1). However, thrombotic events associated with COVID-19 differ from traditional venous thromboembolic disease, in that a high proportion of pulmonary embolism occurrs without deep venous thrombosis of the lower limbs. Autopsy studies described microthrombi in small arteries and pulmonary capillaries. Also in addition to classic thrombosis in the macrocirculation, severe COVID-19 patients could suffer from in situ pulmonary microthrombosis, probably of inflammatory origin, hence the term thrombo-inflammation. The aim of this manuscript is to review hypotheses for the pathophysiology of this severe acute respiratory syndrome leading to thromboembolic events, with the discussion of the intensity of the prophylactic anticoagulation.

### 1.1. D-Dimers and Thromboembolic Events

From the early months of the pandemic, it became evident that the SARS-CoV-2 virus distinguished itself from other viruses due to its notably high morbidity and mortality, particularly in the context of thromboembolic complications. Publications emerging from Wuhan highlighted elevated D-dimer levels among patients upon admission. Both baseline and peak D-dimer levels were found to be closely linked to disease severity, the occurrence of thromboembolic events, transfer to intensive care units and overall mortality [4,5]. However, routine anticoagulant prophylaxis was not universally adopted in the Asian population, considering the relatively low incidence of thromboembolic events in real-life scenarios [6]. In contrast, initial reports from Europe revealed a high frequency of thromboembolic events among hospitalized patients, even in the presence of anticoagulant prevention [5,7]. This was often observed as in situ pulmonary embolism without accompanying deep venous thrombosis, particularly in intensive care units [2,3]. Consequently, it was recommended that prophylactic anticoagulation be considered for all patients requiring hospitalization for COVID-19 [8]. During the initial wave of the pandemic, D-dimer assays were suggested as a means of screening for thrombosis, often with the introduction of a new threshold [9]. This sometimes led to the initiation of high-intensity anticoagulant prophylaxis, with, for example, a cut-off of 3000 µg/L [10]. The incidence of thromboembolic events appeared to decrease during subsequent waves, attributed to improved management strategies, including the administration of corticosteroids and the expanded vaccination efforts.

At the onset of the COVID-19 pandemic, pulmonary embolisms often went unnoticed clinically, resulting in sudden deaths. The initial medical autopsy series played a pivotal role in shedding light on the pathogenesis of this severe infectious disease. As anticipated, massive pulmonary embolism and deep venous thrombosis in the lower limbs were frequently identified. What is noteworthy was that pulmonary embolisms often occurred in situ, remaining undetected during clinical assessments. Histopathological examinations of the lungs revealed a pattern of diffuse alveolar damage, capillary congestion, patchy areas of hemorrhage and the presence of microthrombi within small lung arteries. These findings indicated the involvement of fibrin and platelets within small vessels, suggesting a concurrent microangiopathy. Furthermore, viral RNA was detected not only in the lungs and pharynx but also in the heart, liver, kidney and cerebrum, thus explaining the multi-organ failures observed in severe cases of COVID-19 [11,12].

### 1.2. ACE-2 Receptor and Vascular Injury: The SARS-CoV-2 Vasculopathy

Severe infectious diseases trigger inflammatory responses that are crucial for host defense but can also lead to dysregulated coagulation systems, sometimes resulting in a condition known as disseminated intravascular coagulation (DIC), characterized by the consumption of platelets and coagulation factors. COVID-19 exhibits distinctive features in both the coagulation and inflammatory responses, offering insights into the severity of this disease. COVID-19-associated coagulopathy tends to be more thrombotic in nature compared to coagulopathy induced by sepsis. This is likely a consequence of an intense inflammatory syndrome, stemming from an inappropriate immune response known as the “cytokine storm,” and severe endothelial damage, which collectively lead to a prothrombotic phenotype.

A key player In COVID-19 is the spike protein on the virus, which is composed of two subunits, S1 and S2, that are required for viral entry into cells. The S1 subunit binds the angiotensin-converting enzyme 2 (ACE-2) receptor, and the S2 subunit is cleaved by transmembrane serine protease 2 (TMPRSS2), facilitating the cell entrance of the virus [13]. The SARS-CoV-2 virus initially binds to epithelial cells in the oral cavity via the ACE-2 receptor, resulting in local propagation. In approximately 20% of patients, the virus progresses into the lungs, where it attaches to alveolar cells, leading to severe pulmonary complications that often necessitate mechanical ventilation. While the ACE-2 receptor and TMPRSS2 are predominantly found in the lungs, they are also expressed in various other cell types, including endothelial cells, epithelial cells, cardiomyocytes and enterocytes [14].

Other proteins can facilitate viral entry, such as cathepsin L and furin, but it is the TMPRSS2 pathway that is the fastest. When the TMPRSS2 receptor is absent on the cell surface, the virus takes another way, the endolysosomal pathway, which is slower because it involves multiple cellular mechanisms and an acid PH. This second pathway involves cathepsin L [15]. Notch transmembrane receptors may indirectly promote viral entry via enhanced furin expression. Notch signaling is known to interact with many viral particles (Epstein–Barr virus, Hepatitis B virus, HIV), facilitating their infectivity. An increase in Notch signaling was observed in lungs infected with SARS-CoV-2. Furin is a protease known to cleave the SARS-CoV-2 spike protein [16]. Viral attachment to cells is also facilitated by heparin sulfate [14]. This ACE-2 receptor is already well known in the context of cardiovascular disease, with ACE-2 receptor inhibitors (ARA 2).

The direct action of the SARS-CoV-2 virus on endothelial cells is responsible for severe endothelial damage. Its fixation on the ACE-2 receptor reduces the activity of ACE-2, resulting in increased vascular permeability and the expression of tissue factor (TF). In essence, the severity of COVID-19 is closely linked to severe vasculopathy rather than coagulopathy [17]. Two distinct pathological vascular processes can explain the clinical manifestations. In the systemic circulation, high levels of inflammation (cytokines) and hypercoagulability (elevated levels of fibrinogen, VWF, factor VIII, D-dimer, etc.) predispose individuals to venous or arterial thromboembolic events. Meanwhile, direct vascular and endothelial injury in the microcirculation gives rise to microthrombi and multi-organ dysfunction in severe COVID-19 patients. Systemic complement activation could explain a number of microvascular endothelial injuries, in children in particular [18]. Treating these microthrombi proves to be more challenging than conventional venous and arterial thromboses. A deeper understanding of endothelial damage is imperative to determine the most appropriate therapeutic interventions.

### 1.3. Vasculopathy-Related Coagulopathy

The tropism of SARS-CoV-2 for ACE2 receptors leads to intense endothelial damage, disrupting the natural antithrombotic state. The loss of glycocalyx, which possesses anticoagulant properties, triggers the activation of various coagulation pathways, including the expression of tissue factor, the activation of the contact phase and the disruption of the fibrinolytic system. This cascade ultimately results in the deposition of fibrin in vessels (veins, arteries and arterioles), but also in the microcirculation and pulmonary alveolar spaces via vascular leakage. Alterations in transcriptional factors within endothelial cells, such as HIF-1 (hypoxia-inducible factor 1), could further exacerbate the severity of hypoxemia. Several studies have sought to identify biomarkers of disease progression by comparing patients with pneumonia related to COVID-19 and those without. Endothelial cell adhesion molecules, particularly soluble vascular cell adhesion molecule 1 (sVCAM-1), were significantly associated with the severity of COVID-19, in contrast to coagulation biomarkers. This further supported the notion that vasculopathy is the primary driver of severity in this infectious disease [19,20]. Biomarkers of endothelial damage, including VWF, PAI-1 and TFPI, were independently linked to liver injury and multiorgan failure [21]. Other endothelial biomarkers, such as circulating endothelial cells, soluble thrombomodulin, angiopoietin-2 and E-selectin, were observed at elevated levels in critical COVID-19 patients. However, VWF emerged as the most reliable biomarker for predicting in-hospital mortality [22,23], a finding corroborated through a meta-analysis [24]. A decrease in the FVIII/VWF ratio upon admission was associated with an increased need for oxygen regardless of age, sex, BMI, diabetes and hypertension [25]. The increase in FVIII levels was less pronounced because the liver is only exposed to pro-inflammatory cytokines. It is important to note that VWF release from endothelial cells is stimulated by hypoxia [26], and corticosteroids have been found to decrease endothelial activation and subsequent VWF release.

Vascular activation and injury induce the release of VWF from Weibel–Palade bodies in vascular endothelial cells in large quantities and ultra-large multimer configurations (ULVWFs). These ULVWF forms are cleaved by the ADAMTS 13 protease into smaller forms. In COVID-19, ADAMTS 13 is unable to counterbalance the excess of ULVWFs, resulting in high levels of ULVWFs with pro-aggregating properties. This promotes the formation of in situ microvascular platelet-rich thrombi in the small vessels of the lungs and other organs [22,27].

Note that patients with diabetes, obesity, cardiovascular disease and chronic kidney diseases are at a higher risk of severe COVID-19 because these comorbidities are primarily associated with endothelial dysfunction.

### 1.4. Platelet Activation with Platelet–Leucocyte Aggregates

In most COVID-19 patients, their platelet counts remain within the normal range, and thrombocytopenia (platelet count < 100 G/L) is relatively uncommon, except in intensive care units where there can be multiple contributing factors. However, severe thrombocytopenia was related to a higher risk of death, with approximately half of fatal COVID-19 cases exhibiting severe thrombocytopenia during the first wave [28]. Some studies have shown the presence of viral mRNA in platelets in patients with COVID-19, while certain authors have observed the presence of ACE2 receptors on platelets [29]. However, others have suggested that the absorption of mRNA may occur independently of ACE2 [30]. Platelets from patients with COVID-19 appeared to be hyperactive when exposed to typical agonists like thrombin, ADP and collagen. This activation was associated with elevated levels of sP-selectin and sGPVI. It is worth noting that studies on this topic have produced some controversial results [31,32]. Platelets may play a role in the formation of platelet–monocyte aggregates, leading to the expression of tissue factor by monocytes, the release of procoagulant microparticles and the accumulation of ULVWFs, ultimately resulting in platelet aggregates [33]. Interestingly, pretreatment with aspirin and clopidogrel did not prevent platelet-induced tissue factor expression on monocytes [32,34]. Upon activation, platelets release cytokines that are stored in their granules, which may contribute to the inflammatory response seen in COVID-19. These cytokines include IL-1, IL-10, interferon-α and interferon-δ.

### 1.5. Dysregulation of Immune Response, Cytokine Storm and Complement System Activation

The immune response is a complex network with a multitude of actors and interplays, which guarantees the balance between the production of pro-inflammatory and anti-inflammatory cytokines and the control of the complement system. The severity of COVID-19 is closely linked to an excessive and disproportionate immune response that occurs in some patients after approximately a week of disease progression, commonly referred to as the “cytokine storm.” This phenomenon involves the activation of the complement system and the release of large quantities of cytokines, which activate endothelial cells, exacerbating vasculopathy and endothelial injury and leading to prothrombotic properties. Monocytes and macrophages play a pivotal role in this exaggerated inflammatory response, with high levels of cytokines such as TNF-α, IL-6, IL-8 and IL-1β. Natural killer cells produce a number of cytokines after activation by mediators (IL-6, IFNγ), like epithelial cells and endothelial cells Neutrophils play a vital role by triggering the formation of high amounts of neutrophil extravascular traps (NETs), a network of fibers that contributes to generating a prothrombotic environment through the activation of platelets, the coagulation cascade, the complement system and cytokine and chemokine production [35].

Significantly elevated IL-6 levels were observed in non-survivor COVID-19 patients compared to survivor patients [36]. The efficacy of tocilizumab, a recombinant monoclonal antibody against IL-6, was tested in severe COVID-19 patients, but with discordant results, either a significant reduction in ventilation dependency [37] or without efficacy [38]. Other anti-IL-6 or anti-Il-1β antagonists were studied, but with side effects (neutropenia, liver enzymes). Interestingly, when severe COVID-19 patients were compared to patients with sepsis or other acute respiratory syndromes, no significant differences were observed in terms of their cytokine levels, as reported in a recent meta-analysis [39]. As expected, the severity of COVID-19 is not only explained by cytokine storms, but through interactions between cytokines, the complement system, blood cells, the coagulation cascade and perhaps other agents.

SARS-CoV-2 infection induces lymphopenia, particularly in CD4+ and CD8+ cells, and the reduction in T cells is correlated with the severity of COVID-19. CD4+ T cells play a key role in the immune response, regulating immune cells, especially IFNγ production by CD8+ cells and antibody production by B cells, which are essential for the resistance against infection. The IFNγ response tends to be reduced or delayed in patients with severe COVID-19 [40].

The Notch signaling pathway was explored to explain this inflammation with the cytokine storm. The Notch pathway is a family system of transmembrane receptors, polygenic and multi-ligands that are implicated in the regulation of inflammation and tissue regeneration. Notch signaling has been shown to regulate inflammatory processes in infection, cardiovascular diseases, cancer, etc. These receptors have been reported to promote inflammatory environments by activating macrophages to produce Il-6. But in turn, Il-6 increases Notch signaling, such as TNFα, causing a positive feedback loop that further potentiates cytokine production. It is possible that Notch signaling failed to turn down and stayed elevated in severe COVID-19 patients [41]. The production of pro-inflammatory cytokines involved in the cytokine storm is related to the nuclear activity of NF-κB. The most common and successful treatment targeting NF-kB is dexamethasone, the first-line treatment in cases of severe COVID-19. Dexamethasone is now a standard therapy in hospitalized COVID-19 patients, after the demonstration of a reduction in oxygen therapy and mechanical ventilation requirement, and a reduction in mortality [42]. SARS-CoV-2-infected cells can activate the complement system directly through the lectin pathway. Furthermore, protein spikes may directly dysregulate the alternative pathway by binding to heparin sulfate and competing with inhibitor factor H [16]. High levels of C5a and terminal activation fragments C5b-9 were detected in COVID-19 patients and correlated to clinical severity. The complement system could be particularly implicated in multisystem inflammatory syndrome in children (MISC), a Kawasaki-like disease, with elevated plasma levels of C5b-9 [43]. Diffuse microvascular thrombosis was reported in COVID-19 patients with deposits of complement activation products and fibrin in a histological examination of their lungs, hearts and kidneys, and the presence of hemolysis biomarkers (high levels of LDH, low levels of haptoglobin, schizocytes) could suggest complement-mediated thrombotic microangiopathy [44]. A number of clinical trials have been set up, such as eculizumab (anti-C5a), with a trend for increased survival, but randomized trials are discontinued or ongoing and their potential benefits need to be balanced against the risk of severe infectious complications being added to the COVID-19 disease [16].

### 1.6. Cross-Link between Inflammation and Coagulation

#### 1.6.1. Cytokines, Complement System, NETS and Activation of the Coagulation Cascade

**Figure 2:**
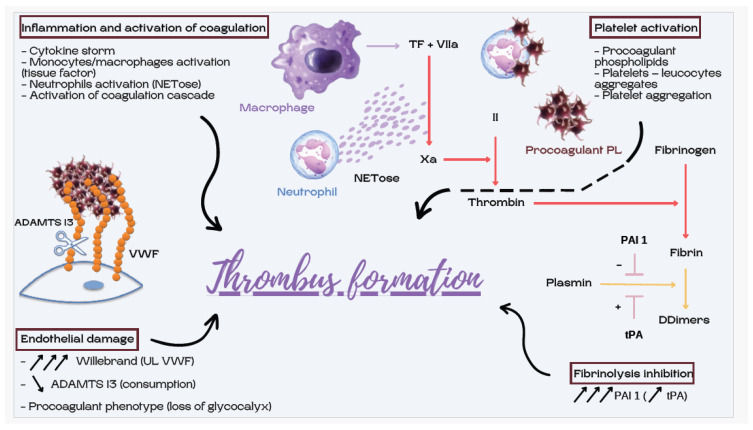
Cross-link between inflammation and coagulation.

The hyperexpression of tissue factor (TF) on monocytes contributes to the activation of the coagulation cascade. The activation of coagulation is associated with the exposure of procoagulant phospholipids, including phosphatidylserine, with the involvement of the scramblase TMEM16F. This flip-flop of phospholipids on the endothelial cell surface may be amplified by the SARS-CoV-2 virus, as an increase in intracellular calcium is required for scramblase activity and phosphatidylserine exposure. The SARS-CoV-2 genome encodes for a cation channel that promotes the action of scramblase [45]. Coagulation is also activated by the inflammatory syndrome through other pathways. Cytokines, particularly interleukin IL-6, induce the liver’s production of coagulation factors, which explains hyperfibrinogenemia (fibrinogen levels > 8–10 g/L). Polyphosphates derived from microorganisms activate platelets and factor XII in the contact pathway of coagulation. NETs are released by recruited neutrophils at a higher density in COVID-19 than in other lung infections, and may contribute to thrombosis. In the lungs, NETs were observed in association with platelets, VWF, tissue factor and fibrin, suggesting that NETs could activate the coagulation cascade [46]. The colocalization of NETs with factor XII was confirmed, and NETs could be a platform for the activation of the intrinsic coagulation pathway (factor XII) [47]. Activated complement components can promote cell activation, leading to hypercoagulability: C5a recruits and activates neutrophils, monocytes and macrophages, and promotes platelet adhesion, the expression of adhesion molecules on endothelial cells, and C3a recruits and activates neutrophils releasing NETs [16] (Figure 2).

#### 1.6.2. CAC Rather Than DIC

Interestingly, this activation of the coagulation cascade does not lead to the typical disseminated intravascular coagulation (DIC) seen in sepsis, as there is no consumption of fibrinogen and platelets, and the International Society on Thrombosis and Haemostasis (ISTH) criteria for DIC are most often negative. Conversely, very high levels of fibrinogen (up to 10–12 g/L) were observed, and thrombocytopenia was rare and typically moderate. Nevertheless, high levels of D-dimers in critically ill patients indicated an intense hypercoagulable state, which was responsible for fibrin deposition and thrombotic events, including venous thromboembolic events in 25–35% of cases and arterial thrombosis in 1–5% of cases (such as ischemic stroke, acute coronary syndrome and systemic arterial embolism). Instead of DIC, the term “CAC” (coagulopathy associated with COVID-19) has been proposed [48]. Presence of antiphospholipid antibodies was detected (especially lupus anticoagulant) in the acute phase of COVID-19, with a potential implication in the occurrence of thromboembolic complications, but this was not later confirmed (transient antibodies, rather related to infection) [49].

#### 1.6.3. Impaired Fibrinolysis

In the acute inflammatory state, a complex interplay of fibrinolysis occurs, starting with a transient increase in tissue plasminogen activator (tPA), rapidly followed by the sustained release of inhibitors of tPA, such as plasminogen activator inhibitor 1 (PAI-1) and thrombin-activatable fibrinolysis inhibitor (TAFI). This dynamic process eventually leads to a transition from hyperfibrinolysis to hypofibrinolysis. In the context of COVID-19, the hypothesis of impaired fibrinolysis was explored. The increase in PAI-1 and TAFI was more pronounced than the increase in tPA, resulting in a hypofibrinolytic state [50,51]. The overexpression of PAI-1 was observed in the lungs of post-mortem COVID-19 cases, with staining for PAI-1 found in epithelial cells, macrophages and endothelial cells, creating a local hypofibrinolytic environment [51]. High levels of PAI-1 were detected in bronchoalveolar lavage fluid collected from critically ill COVID-19 patients, and impaired fibrinolysis in the lungs may contribute to the in situ deposition of fibrin. A multivariate analysis has shown that PAI-1 levels predict both mortality and disease severity in COVID-19 [52]. Notably, PAI-1 is also produced by adipose tissue and is elevated in obese patients. Obesity is a comorbidity associated with poor outcomes in COVID-19 infections.

#### 1.6.4. D-Dimers and Fibrin Monomers

**Figure 3:**
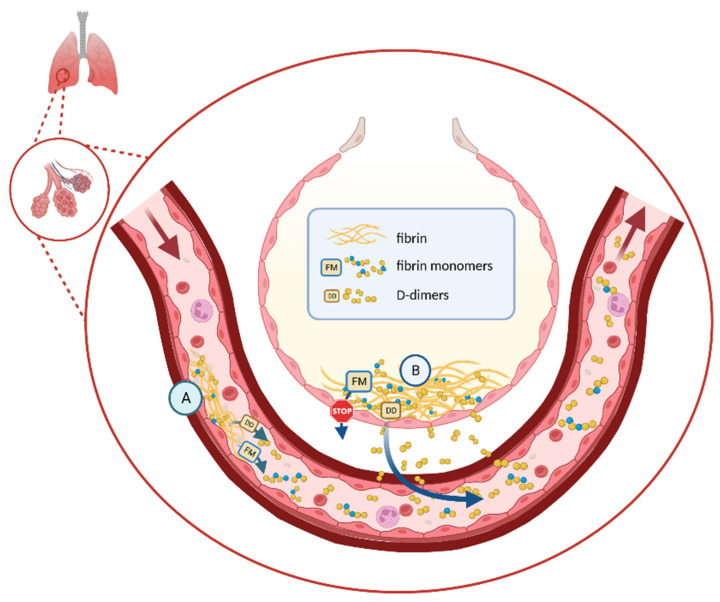
Distinction between D-dimers (DD) with a low MW allowing extravasation (A and B) and a fibrin monomer (FM) with a high MW (A); MW: molecular weight; A: intravascular area; B: extravascular area (pulmonary alveolar). (Created with Biorender.com, accessed on 31 October 2023).

The monitoring of D-dimer levels every 48 h was suggested to serve as an alert for possible thromboembolic events or to guide decisions on the adjustment of anticoagulation intensity, considering bleeding risks [9,53]. However, it is important to note that the precise threshold values for D-dimers in these specific clinical situations need to be established and may vary between different D-dimer immunoassays, particularly in cases of high D-dimer levels due to the heterogeneity of fibrin degradation products [54]. D-dimers may not be the optimal biomarker since their concentration increases secondarily to clot lysis and can be influenced by both intravascular and extravascular fibrin deposits. Indeed, in post-mortem examinations, extravasated erythrocytes and a loose network of fibrin were observed in the intra-alveolar space. As an alternative, the measurement of fibrin monomers (or soluble fibrin complexes) was proposed in COVID-19 patients because they are specific markers of intravascular thrombin generation. These molecules have a higher molecular weight than D-dimers due to the assembly of fibrin monomers with fibrinogen molecules, preventing their exit into the extravascular space (Figure 3). In the absence of thrombosis or DIC/CAC, fibrin monomers were often low in COVID-19 patients (contrary to D-dimers) and it could be easier to detect an abrupt rise related to a thrombotic event [55]. Moreover, the peaks were often transient, and the levels decreased more rapidly than those of D-dimers [56]. The short half-life of fibrin monomers requires daily dosing to detect these abrupt hypercoagulability states [57].

### 1.7. Prevention for Thrombotic Risk in Hospitalized Patients: Escalation of Anticoagulant Intensity?

Early reports from European intensive care units highlighted a higher incidence of thromboembolic events in severe COVID-19 patients compared to historical cases of severe infectious diseases, despite standard anticoagulant prophylaxis [1,2,3,7]. Several factors may have contributed to this increased risk, including severe endothelial injury exacerbated by hypoxia and the need for mechanical ventilation, hypercoagulability, obesity and personal or familial risk factors. This apparent rise in thromboembolic events among COVID-19 patients prompted rapid decisions by experts and scientific societies, even before the availability of clinical trial results [58]. Many medical centers increased the dosage of anticoagulation for prophylaxis to intermediate the intensity (or doubled the dose) and, in some cases, to therapeutic levels using algorithms based on clinical factors (intensive care unit admission, BMI, oxygen requirements) and/or laboratory data (D-dimers, fibrinogen levels) [9]. The decision to use therapeutic doses was also motivated by the desire to reduce sudden deaths from presumed pulmonary embolism, as well as the challenges of performing CT scans in mechanically ventilated patients, but high doses could expose patients to bleeding complications.

Three years later, the need to escalate anticoagulation dosages is not as clear-cut. Several prospective studies have supported the benefits of high-dose prophylactic anticoagulation (either intermediate or therapeutic) in critically ill and even non-critically ill patients [59]. However, these results have not been consistently observed in randomized studies using intermediate doses (e.g., the INSPIRATION study) [60] or therapeutic doses (e.g., the HEP-COVID, ATTACC, ACTIV-4a and CEMAP-CAP studies) [61,62,63,64]. The ISTH guidelines published in 2022 recommended prophylactic doses of low-molecular-weight heparin (LMWH) and unfractionated heparin (UFH) in non-critically ill patients, as well as critically ill patients, with exceptions for select patients at a high risk of thrombosis [65]. Following the first wave of the pandemic, the incidence of thromboembolic events decreased due to advancements in patient management, including the use of corticosteroids and immunomodulatory treatments. Corticosteroids, such as dexamethasone, have been a key intervention in managing severe COVID-19 cases. These drugs effectively modulate systemic inflammation by suppressing the immune response and reducing the production of pro-inflammatory cytokines. By dampening the “cytokine storm,” corticosteroids may indirectly lower the risk of vasculopathy and thrombosis. The decreased prevalence of thrombosis observed in later stages of the pandemic may be attributed, in part, to the systemic anti-inflammatory effects of corticosteroid treatment.

Recently, a multicenter randomized study (the COVIDOSE study) confirmed that prophylactic doses of LMWH were sufficient to control the risk of symptomatic thromboembolic events, and that higher doses were associated with a twofold increased risk of clinically relevant non-major bleeding [66]. Critically ill patients were often exposed to bleeding events that could be life-threatening.

Antithrombotic treatments other than heparins were studied in COVID-19. Taking into account the heparin resistance associated with severe inflammatory syndrome, direct oral anticoagulants (DOACs) were studied, but their half-life (around 12 h) is not adapted in intensive care units. Parenteral anti-thrombin anticoagulants such as argatroban were also tested, with difficulties in determining the optimal dose (limited experience outside heparin induced thrombocytopenia) and the underlying hemorrhagic risk without an antidote [67]. An abundance of fibrin deposition in the pulmonary alveolar capillaries and the relative hypofibrinolysis led to discussions on the the use of tPA, in particular nebulized tPA, to promote fibrin dissolution in the lungs, but without positive results [68]. With the complexity of this hypercoagulable state, with a major role played by endothelial damage, the picture could be compared to that of veno-occlusive disease (VOD), hence the idea of using defibrotide, the first drug approved in the US for the treatment of hepatic VOD. Defibrotide is known not only for its profibrinolytic activity, but also for its anticoagulant and endothelial-protective action (anti-inflammatory and antioxidant effects) [69,70]. In a prospective study, the use of defibrotide was safe (no hemorrhagic or thrombotic complications), with encouraging results (improvement in respiratory functions and biological parameters). However, only 12 patients were included, and the association of defibrotide with heparin is normally contraindicated [71].

This review is subject to several limitations. Firstly, our selection of studies and hypotheses was drawn from the extensive pool of COVID-19 publications (currently totaling 394,380 results on PubMed). Additionally, the SARS-CoV-2 virus exhibits a high mutation rate, a characteristic common among RNA viruses, potentially leading to significant implications in physiopathology.

## 2. Conclusions

COVID-19 indeed illustrates the components of the Virchow triad, encompassing coagulopathy, venous stasis (often attributed to bed rest in critically ill patients) and endothelial damage. At present, there exists no specific antiviral therapy tailored for SARS-CoV-2 infection. The treatment primarily revolves around managing acute lung injuries and acute respiratory distress syndrome, aligning with the standard approach for critically ill septic patients. A multitude of prospective and retrospective studies have been undertaken to deepen our understanding of COVID-19’s pathophysiology and enhance therapeutic strategies, especially concerning anticoagulation.

Fortunately, the severity of COVID-19 infections has diminished with the advent of the latest SARS-CoV-2 variants and widespread post-COVID-19 immunization and vaccination efforts. These advancements have played a pivotal role in mitigating the overall impact and seriousness of the disease. However, it is imperative that we draw lessons from the COVID-19 pandemic. Experts are already cautioning us about the potential for future pandemics in the years ahead. We must strive to share information more swiftly and, most importantly, more openly and transparently. This collaborative approach is crucial for gaining deeper insights into how viruses emerge, enabling us to optimize patient care and prevent drastic measures like population-wide lockdowns, with all the economic and mental health consequences they entail. It is vital for everyone to remain vigilant, adhering to basic hygiene measures such as regular handwashing with hydro-alcoholic gel and wearing masks, especially in healthcare settings, to prevent nosocomial infections.

## Data Availability

Not applicable.
